# Complete genome sequence of strain JHJ, a chloromethane-degrading anaerobe affiliated with the family *Oscillospiraceae*

**DOI:** 10.1128/mra.00983-25

**Published:** 2025-11-24

**Authors:** Huijuan Jin, Yi Yang, Ke Shi, Xiuying Li, Jingjing Wang, Yiru Cui, Siqi Huang, Tongyue Zhou, Guanchi Liu, Gao Chen, Frank E. Löffler, Jun Yan

**Affiliations:** 1 Key Laboratory of Pollution Ecology and Environmental Engineering, Institute of Applied Ecology, Chinese Academy of Sciences 74763 , Shenyang, Liaoning, China; 2 University of Chinese Academy of Sciences, Beijing, China; 3 College of Life Sciences, Shenyang Normal University 12402, Shenyang, Liaoning, China; 4 Department of Civil and Environmental Engineering, University of Tennessee 4292, Knoxville, Tennessee, USA; 5 Department of Biosystems Engineering and Soil Science, University of Tennessee 4292, Knoxville, Tennessee, USA; 6 Department of Biochemistry & Cellular and Molecular Biology, University of Tennessee 4292, Knoxville, Tennessee, USA; University of Southern California, Los Angeles, California, USA

**Keywords:** chloromethane, anaerobic fermentation, formate, estuarine environment

## Abstract

*Oscillospiraceae* bacterium strain JHJ, an anerobe isolated from estuarine sediment, degrades chloromethane to formate and acetate. The genome of strain JHJ consists of a 2.2 Mbp circular chromosome with a G + C content of 52.5% and encodes an incomplete Wood-Ljungdahl pathway and 52 putative methyltransferases.

## ANNOUNCEMENT

Chloromethane (CM) is Earth’s most abundant halocarbon, primarily emitted from vegetation, soils, and marine environments (1, 2). Despite the impact on ozone depletion and climate change, the biotransformation and environmental fate of CM under anoxic conditions remains poorly understood (3). Sediment was collected from Wuli River Estuary in Huludao, Liaoning, China (40°44′44″ N, 120°59′11″ E) using an alcohol-sterilized shovel, placed in a 1 L sealable bag, and transported to the laboratory on ice. An anaerobic consortium derived from the sediment sample was cultivated at 30°C in 160 mL serum bottles containing 100 mL reduced mineral salts medium (4) under a N_2_/CO_2_ (80/20, vol/vol) headspace, and completely converted 89.3 µmol CM to formate and acetate within 7 days (5). Dilution-to-extinction in semi-solid (0.8% low-melting agarose) medium (4) supplemented with CM yielded uniform white colonies, from which a strictly anaerobic bacterium was obtained. This isolate, now designated *Oscillospiraceae* bacterium strain JHJ, was previously referred to as *Sporobacter* sp. strain CM (5).

Following the consumption of 4.5 mmol CM over 2 weeks, biomass was harvested from a 1.6 L liquid strain JHJ culture grown in the reduced mineral salts medium (4) under the conditions described above. The same genomic DNA extracted using the CTAB protocol (6) served as the template for both Illumina and Nanopore library preparations. Illumina libraries were constructed with the NEBNext Ultra DNA Library Prep Kit (New England Biolabs, Ipswich, MA) and sequenced (PE150) on a NovaSeq 6000 platform (Illumina, San Diego, CA). For Nanopore sequencing, ~10 kb DNA fragments were size-selected using a BluePippin system (Sage Science, Beverly, MA), end-repaired, barcoded with the EXP-NBD104 Kit, and sequenced on a PromethION platform (R10.4.1 flow cell) using the ligation (1D) workflow. Base calling was performed in high-accuracy mode with Guppy v6.4.6 (Nanopore Technologies, Oxford, UK). Raw reads were quality-filtered using fastp v0.20.0 (7), generating 6,339,596 short reads (*Q*_30_ = 94.2%) and 78,136 long reads (*N*_50_ = 15,165 bp). *De novo* genome assembly was performed using Unicycler v0.48 (8). Functional annotation and genome re-orientation to *dnaA* were performed using the NCBI Prokaryotic Genome Annotation Pipeline (9). Average nucleotide identity (ANI) was calculated using JSpeciesWS v4.1.1 (10). Default settings were used for bioinformatics analyses unless otherwise stated.

Strain JHJ genome was assembled at 325.0× coverage, yielding a single circular 2,185,546 bp chromosome with a G + C content of 52.5%. Genome annotation revealed 2,087 protein-coding genes, 48 tRNAs, two copies of 5S-16S-23S rRNAs, and an incomplete Wood-Ljungdahl pathway lacking the formate dehydrogenase gene (*fdh*). The genome also features 52 putative methyltransferase genes, suggesting strong adaptation to methylated substrates including CM. Strain JHJ is distantly related to *Sporobacter termitidis* DSM 10068 (FQXV00000000), with sequence similarities near the genus-level threshold (i.e., 95.4% 16S rRNA gene identity and 72.1% ANI). Notably, *S. termitidis* DSM 10068 has a larger genome of 4.2 Mbp (11). These lines of evidence suggest that strain JHJ represents a new lineage within the family *Oscillospiraceae*. The isolation of strain JHJ expands the diversity of CM-degrading phylotypes and provides new genomic insights into microbial CM turnover in anoxic environments.

## Data Availability

The complete sequence of strain CM genome has been deposited at GenBank under accession number CP072107. The BioSample and BioProject accession numbers are SAMN16807137 and PRJNA678572, respectively. Genome sequencing raw reads were deposited in the Sequence Read Archive (SRA) under accession numbers SRR35137325 (Illumina) and SRR35136564 (Nanopore).
